# Individual time course of pre- and postsynaptic PET imaging may improve differential diagnosis of Parkinson’s disease and multiple system atrophy: a case report

**DOI:** 10.1186/s13104-015-1522-0

**Published:** 2015-09-29

**Authors:** Kenji Ishibashi, Hirofumi Nishina, Kiichi Ishiwata, Kenji Ishii

**Affiliations:** Research Team for Neuroimaging, Tokyo Metropolitan Institute of Gerontology, 35-2 Sakae-cho, Itabashi-ku, Tokyo, 173-0015 Japan; Department of Neurology, Tokyo Metropolitan Geriatric Hospital, Tokyo, 173-0015 Japan

**Keywords:** Parkinson’s disease, Multiple system atrophy, Dopamine transporter, Dopamine D_2_ receptor, Positron emission tomography

## Abstract

**Background:**

Many in vivo studies have shown a difference in pre- and/or postsynaptic imaging between Parkinson’s disease and multiple system atrophy; however, time course differences in pre- and postsynaptic imaging between Parkinson’s disease and multiple system atrophy have not been rigorously investigated.

**Case presentation:**

We report serial positron emission tomography images of both dopamine transporters and dopamine D_2_ receptors, obtained from a Japanese patient with Parkinson’s disease who underwent positron emission tomography scanning at ages 71, 72, 74, and 75 years, and another Japanese patient with multiple system atrophy who underwent positron emission tomography scanning at ages 65, 66, and 67 years. Volumes-of-interest were placed on the striatal subregions. The percentage decreases between the first and last images showed that dopamine transporter availability decreased with disease progression in both patients, but that dopamine D_2_ receptor availability decreased only in the patient with multiple system atrophy. A partial correlation analysis between dopamine transporter and dopamine D_2_ receptor availability, controlling for the effects of striatal subregional differences, revealed a positive correlation in the patient with multiple system atrophy (r = 0.893, *P* = 0.0002), but no significant correlation in the patient with Parkinson’s disease (r = −0.036, *P* = 0.89).

**Conclusions:**

The time course of pre- and postsynaptic imaging can be considerably different between Parkinson’s disease and multiple system atrophy, and may be useful in improving the accuracy of discrimination between Parkinson’s disease and multiple system atrophy.

## Background

Parkinson’s disease (PD) and multiple system atrophy (MSA) are neurodegenerative disorders affecting the nigrostriatal dopaminergic system. Imaging of presynaptic neurons using positron emission tomography (PET) or single photon emission computed tomography (SPECT) with a radioligand for dopamine transporters (DATs) cannot distinguish between PD and MSA, especially on the individual level, because both diseases cause neuronal degeneration in the substantia nigra (SN) [1, 2]. However, PET or SPECT imaging of postsynaptic neurons with a radioligand for dopamine D_2_ receptors (D_2_Rs) can improve the accuracy of discrimination between PD and MSA, as D_2_R-expressing striatal neurons tend to degenerate in MSA but not in PD [3, 4].

Many in vivo studies have shown a difference in pre- and/or postsynaptic imaging between PD and MSA [5–8]; however, differences in the time course of these measures between PD and MSA have not been rigorously investigated. In order to address this deficit, we examined pre- and postsynaptic imaging time courses in a patient with PD and another with MSA, focusing on the differential diagnosis between PD and MSA. To quantify availability of DATs and D_2_Rs, we used carbon-11-labeled 2β-carbomethoxy-3β-(4-fluorophenyl)-tropane (^11^C-CFT) and carbon-11-labeled raclopride (^11^C-raclopride), respectively.

## Case presentation

### Research participants

Participants comprised two Japanese female patients, one each with PD and MSA, who were recruited from cross-sectional studies of PD and PD-related disorders at the Tokyo Metropolitan Institute of Gerontology [9]. The PET data used in this study were collected for research purposes. All procedures were approved by the Ethics Committee of the Tokyo Metropolitan Institute of Gerontology. The two patients provided written informed consent for publication.

### Patient with Parkinson’s disease

Six months after developing a right leg tremor, she was diagnosed with PD at the age of 71, and underwent ^11^C-CFT and ^11^C-raclopride PET scanning at ages 71, 72, 74, and 75 years. On initial examination at age 71, she had resting tremor and mild rigidity of her right arm and leg. Levodopa was administered and effective, but without levodopa a mild postural instability developed at age 74. Her Hoehn and Yahr stage at ages 71 and 75 were 1 and 3, respectively.

### Patient with multiple system atrophy

A 65-year-old woman was referred to the neurology department of our hospital after she had developed a progressive gait disturbance over the previous 2 years. On initial examination, she had parkinsonian symptoms (postural instability, bradykinesia, and mild cogwheel rigidity on the left side), cerebellar ataxia, pyramidal sign in both legs, and orthostatic hypotension which was confirmed by a head-up tilt test. She was diagnosed with MSA, and underwent ^11^C-CFT and ^11^C-raclopride PET scanning at ages 65, 66, and 67 years. Levodopa was ineffective. Ages for aid-requiring walking, wheelchair dependence, and a bedridden state were 65, 66, and 67 years, respectively.

### Positron emission tomography scanning and data analysis

PET scanning was performed on a SET-2400 W scanner (Shimadzu, Kyoto, Japan) in three-dimensional mode at the Institute. Static emission data were acquired for 75–90 min and 40–55 min after an intravenous bolus infusion of ^11^C-CFT and ^11^C-raclopride, respectively. The injection doses for both radioligands were 300 MBq.

Volumes-of-interest (VOIs) were placed on the striatal subregions: the ventral striatum (VST), pre-commissural dorsal caudate (pre-DCA), post-commissural caudate (post-CA), pre-commissural dorsal putamen (pre-DPU) and post-commissural putamen (post-PU) [10]. A visual cortex VOI was also created and used as a reference region [11, 12]. To estimate DAT and D_2_R availability in each VOI, the uptake ratio index (URI) of ^11^C-CFT and ^11^C-raclopride was calculated by the following formula: URI = [(activity in the target region) − (activity in the visual cortex)]/[(activity in the visual cortex)] [12]. The normal range for URI is different among striatal subregions. In order to compare the magnitude of changes in DAT and D_2_R availability from the first to last PET images across striatal subregions, the Z score for each was calculated in contrast with the mean and standard deviation (SD) values of controls (with data belonging to the Tokyo Metropolitan Institute of Gerontology), as in the following formula: Z score = [(URI in the patient) − (mean URI in controls)]/[(SD URI in controls)].

In order to compare the time course of pre- and postsynaptic imaging between the patients with PD and MSA, we tested the relationship between DAT and D_2_R availability with a partial correlation approach, controlling for the effects of striatal subregional differences, using SPSS Statistics version 22 (IBM, Armonk, NY). Statistical significance was set at *P* < 0.05.

### Results

**Fig. 1:**
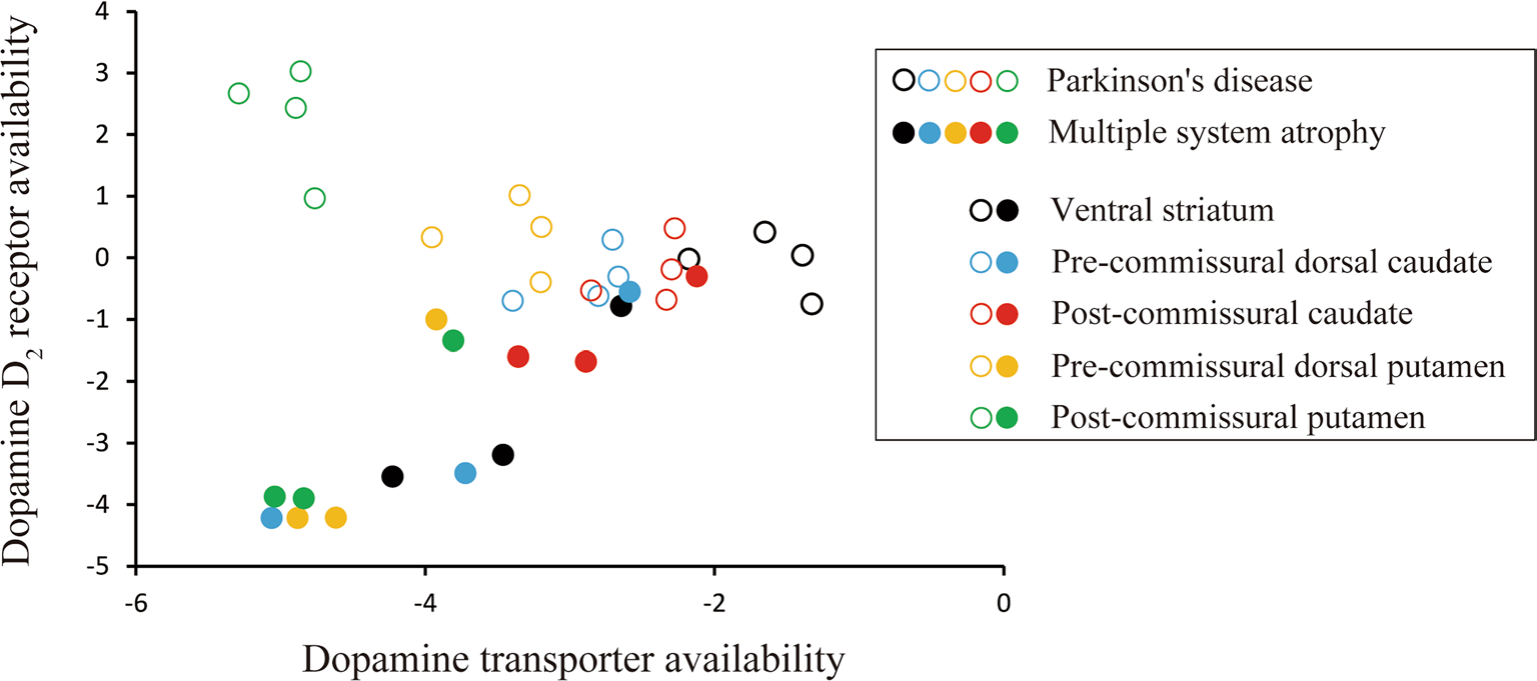
Comparison of the time course of dopamine transporter and dopamine D_2_ receptor availability between the patient with Parkinson’s disease and the patient with multiple system atrophy. Data in all striatal subregions from the first to last images were used. The horizontal and vertical axes represent the Z scores of dopamine transporter and dopamine D_2_ receptor availability, respectively. Black, blue, red, yellow, and green circles represent data from the ventral striatum, pre-commissural dorsal caudate, post-commissural caudate, pre-commissural dorsal putamen, and post-commissural putamen, respectively. Open and closed circles represent the patient with Parkinson’s disease and multiple system atrophy, respectively. The partial correlation, controlling for the effects of regional differences, is significant in the patient with multiple system atrophy (r = 0.893, *P* = 0.0002), but not in the patient with Parkinson’s disease (r = −0.036, *P* = 0.89)

The Z score and percentage decreases of DAT and D_2_R availability from the first to last PET images in each striatal subregion and whole striatum are shown in Fig. 1 and Table 1, respectively. Table 1 shows that DAT availability decreased with disease progression in both patients, and that D_2_R availability also decreased in the patient with MSA, but not the patient with PD. The partial correlation analysis showed a positive correlation between DAT and D_2_R availability for the patient with MSA (partial correlation coefficient: r = 0.893, *P* = 0.0002), but there was no any significant correlation for the patient with PD (r = −0.036, *P* = 0.89).

**Table 1:** Percentage decreases of dopamine transporter and dopamine D_2_ receptor availability between the first and last positron emission tomography images

	Ventral striatum (%)	Pre-commissural dorsal caudate (%)	Pre-commissural dorsal putamen (%)	Post-commissural caudate (%)	Post-commissural putamen (%)	Whole striatum (%)
Parkinson’s disease						
Dopamine transporter	17.4	18.0	21.2	31.7	28.1	21.3
Dopamine D_2_ receptor	−14.6	1.5	−9.7	−5.4	−22.6	−13.3
Multiple system atrophy						
Dopamine transporter	44.6	70.5	34.1	66.8	43.7	50.7
Dopamine D_2_ receptor	55.6	67.7	46.4	41.3	48.7	53.3

Percentage decreases were calculated by the following formula: 100 × [(uptake ratio index in the first image) − (uptake ratio index in the last image)]/(uptake ratio index in the first image)

**Fig. 2:**
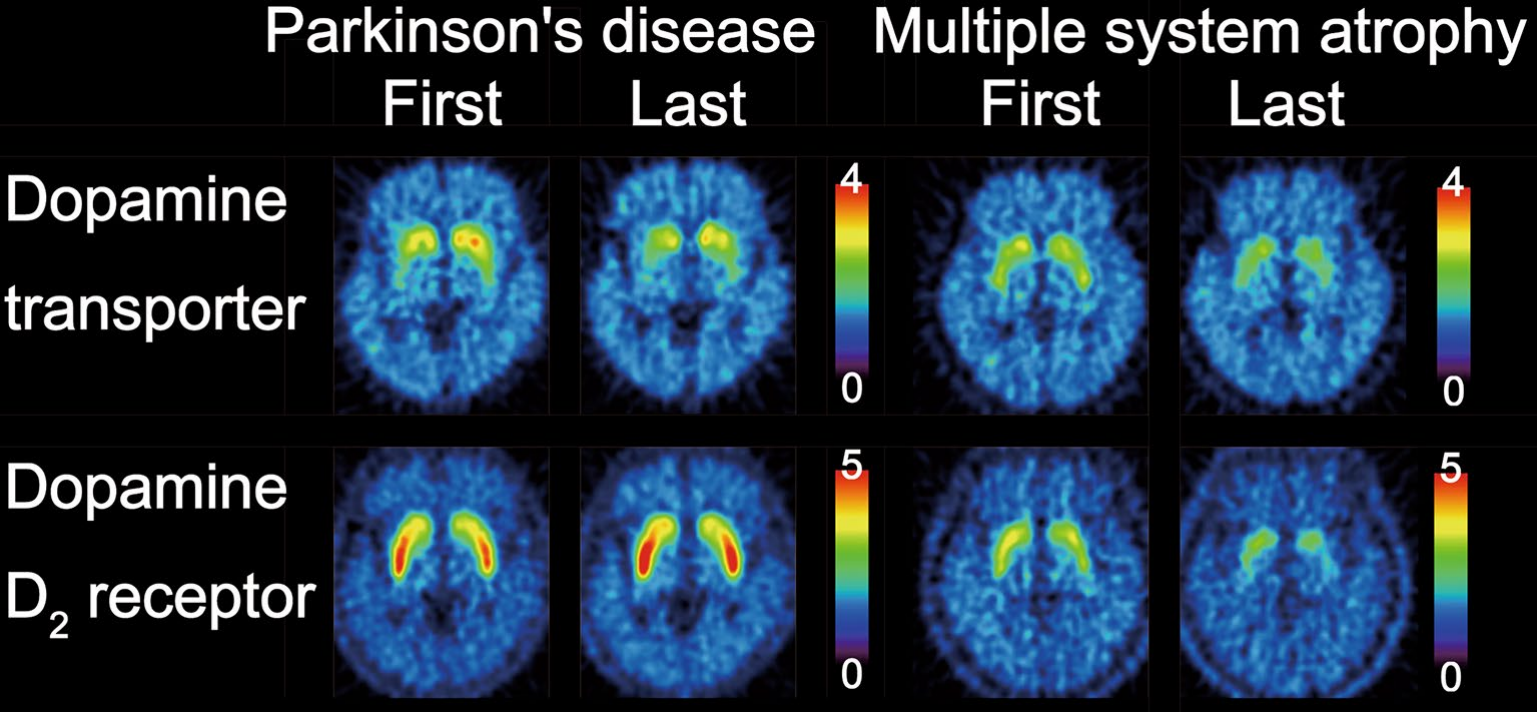
Dopamine transporter and dopamine D_2_ receptor images. The first and last images of dopamine transporters and dopamine D_2_ receptors in the patient with Parkinson’s disease and the patient with multiple system atrophy are displayed in axial sections. The rainbow scale represents the magnitude of uptake ratio index

The first and last DAT and D_2_R PET images are displayed in Fig. 2. Consistent with Table 1, DAT availability tended to decrease in both patients. However, D_2_R availability tended to decrease in the patient with MSA, but not the patient with PD.

## Discussion and conclusions

Longitudinal changes in DAT and D_2_R imaging have been established in PD patents. The dorsal posterior part of the putamen, which is anatomically equivalent to the post-PU in this study, is the initial region of DAT loss and is most severely affected throughout the illness [2, 9, 10]. The number of DATs in each subregion of the striatum decreases along with neuronal loss in the SN and the disease progression, presumably following an exponential curve [2, 10, 13]. On the other hand, according to in vitro studies showing that the number of striatal D_2_Rs is constant even in the advanced stage of PD [3, 4], striatal D_2_R availability in an imaging study is also expected to be constant throughout the illness. D_2_R images, however, may be complicated. In early PD, the expression of D_2_R may be upregulated as a compensatory response to a decrease in endogenous dopamine levels [14, 15]. In longstanding PD, chronic dopaminergic therapy or structural adaptation of the postsynaptic neurons to the progressive degeneration of the presynaptic dopaminergic system may downregulate D_2_R expression [16]. Additionally, the loss of endogenous dopamine can cause increased binding of ^11^C-raclopride (i.e., increased D_2_R availability) throughout the illness [17]. Our findings from an early PD patient were explainable based on those findings.

To our knowledge, this is the first study to investigate the time course of pre- and postsynaptic imaging in a patient with MSA, and that showed a positive correlation between the two (Fig. 1). A pathological study with 35 MSA patients has reported that neurons in both the SN and striatum, especially the putamen, were severely depleted in most cases, and that the degree of nigral and putaminal damage tended to be associated with disease duration [1]. The latter finding was supported by two neuroimaging studies (not longitudinal) showing a positive correlation between DAT and D_2_R availability in MSA groups [6, 12]. Together, previous and our findings show that the functional impairment of both pre- and postsynaptic dopaminergic systems in MSA should develop with disease progression due to neuronal degeneration in the SN and striatum.

Differentiating MSA from PD is often difficult in their early stages, and this sometimes remains difficult even in the late stages of these diseases. About 20 % of patients still carrying a diagnosis of PD were pathologically diagnosed with another neurodegenerative disorder at the time of death, and MSA was the most frequently misidentified pathology [18, 19]. Pre- and postsynaptic striatal imaging can help in differential diagnosis; however, not all MSA patients exhibit a significant reduction of D_2_R availability [20], especially when neuronal loss in the striatum is mild. DAT imaging can demonstrate differences between PD and MSA on the group level but not on an individual level [7]. On the other hand, the present study showed that along with decreasing DAT availability (i.e., disease progression), D_2_R availability also decreased in the patient with MSA, but not in the patient with PD, and suggests that the time course of pre- and postsynaptic imaging can be considerably different between these conditions. Thus, in cases where initial pre- and postsynaptic imaging cannot discriminate between PD and MSA, observation of the individual time courses of pre- and postsynaptic imaging, at an interval of at least a few years, may improve the differential diagnosis of PD and MSA. However, when pre- and postsynaptic imaging is used for clinical purposes, the subsequent PET or SPECT scans should be reserved for unclear cases and their indication should be scrutinized to avoid cumulative exposure to radiation.

One of the limitations of the present study is that PD and MSA are heterogeneous disorders with multiple factors contributing to symptoms and disease progression [21, 22]. Thus, one cannot necessarily expect other patients with PD or MSA to follow the cases presented here. However, as typical cases of PD and MSA, our cases may provide essential information for differentiating between PD and MSA.

## Abbreviations

PD: Parkinson’s disease
MSA: multiple system atrophy
DAT: dopamine transporter
D_2_R: dopamine D_2_ receptor
PET: positron emission tomography
SPECT: single photon emission computed tomography
SN: substantia nigra
^11^C-CFT: carbon-11-labeled 2β-carbomethoxy-3β-(4-fluorophenyl)-tropane
^11^C-raclopride: carbon-11-labeled raclopride
VOI: volume-of-interest
VST: ventral striatum
pre-DCA: pre-commissural dorsal caudate
post-CA: post-commissural caudate
pre-DPU: pre-commissural dorsal putamen
post-PU: post-commissural putamen
URI: uptake ratio index
SD: standard deviation
